# Efficient Degradation of Sulfamethoxazole by Diatomite-Supported Hydroxyl-Modified UIO-66 Photocatalyst after Calcination

**DOI:** 10.3390/nano13243116

**Published:** 2023-12-11

**Authors:** Hui-Lai Liu, Yu Zhang, Xin-Xin Lv, Min-Shu Cui, Kang-Ping Cui, Zheng-Liang Dai, Bei Wang, Rohan Weerasooriya, Xing Chen

**Affiliations:** 1 Key Laboratory of Nanominerals and Pollution Control of Higher Education Institutes, School of Resources and Environmental Engineering, Hefei University of Technology, Hefei 230009, China; 18255110208@163.com (H.-L.L.); 18691965261@163.com (Y.Z.); lvx1996@163.com (X.-X.L.); mcui@mail.ustc.edu.cn (M.-S.C.); cuikangping@hfut.edu.cn (K.-P.C.); 2 Key Lab of Aerospace Structural Parts Forming Technology and Equipment of Anhui Province, Institute of Industry and Equipment Technology, Hefei University of Technology, Hefei 230009, China; rohan.we@nifs.ac.lk; 3 Anqing Changhong Chemical Co., Ltd., Anqing 246002, China; 18256240803@163.com (Z.-L.D.); 13855694589@163.com (B.W.); 4 National Centre for Water Quality Research, National Institute of Fundamental Studies, Hantana, Kandy 20000, Sri Lanka

**Keywords:** diatomite, HO-UIO-66/DE-300/H_2_O_2_/light process, photo-Fenton catalyst, sulfamethoxazole

## Abstract

Sulfamethoxazole (SMX) is a widely used antibiotic to treat bacterial infections prevalent among humans and animals. SMX undergoes several transformation pathways in living organisms and external environments. Therefore, the development of efficient remediation methods for treating SMX and its metabolites is needed. We fabricated a photo-Fenton catalyst using an UIO-66 (Zr) metal–organic framework (MOF) dispersed in diatomite by a single-step solvothermal method for hydroxylation (HO-UIO-66). The HO-UIO-66-0/DE-assisted Fenton-like process degraded SMX with 94.7% efficiency; however, HO-UIO-66 (Zr) is not stable. We improved the stability of the catalyst by introducing a calcination step. The calcination temperature is critical to improving the catalytic efficiency of the composite (for example, designated as HO-UIO-66/DE-300 to denote hydroxylated UIO-66 dispersed in diatomite calcined at 300 °C). The degradation of SMX by HO-UIO-66/DE-300 was 93.8% in 120 min with 4 mmol/L H_2_O_2_ at pH 3 under visible light radiation. The O1s XPS signatures signify the stability of the catalyst after repeated use for SMX degradation. The electron spin resonance spectral data suggest the role of h^+^, •OH, •O_2_^−^, and ^1^O_2_ in SMX degradation routes. The HO-UIO-66/DE-300-assisted Fenton-like process shows potential in degrading pharmaceutical products present in water and wastewater.

## 1. Introduction

Sulfamethoxazole (SMX) is an effective bactericidal sulfonamide drug widely used to combat urinary tract infections and bronchitis. Sulfonamides undergo various metabolic pathways in organisms, which excrete active and inactive metabolites into the environment. Due to the abuse and discharge, SMX is frequently detected in aquatic environments, including rivers (2–630 ng/L), lakes (10–60 ng/L), groundwater (10–250 ng/L), and oceans (3–140 ng/L) [1]. It is noteworthy that currently, there are no maximum permissible concentrations regulated by law for pharmaceuticals [2]. SMX and its metabolites are ubiquitous in surface and wastewater, and they readily dissolve and are not biodegradable. SMX (and other sulfonamides) in animal husbandry as a growth promoter was banned in 2006. However, the persistence of SMX and its metabolites in the environment did not decline [3]. Some metabolites of SMX such as N^4^–acetyl sulfamethoxazole can retransform into the parent compound [4,5,6]. 

Many remedial methods are available to destroy SMX and its metabolites in water and wastewater. The traditional techniques based on adsorption are not efficient in this context. Advanced oxidation processes such as electrochemical oxidation, the Fenton-like process, photocatalysis, or hybrid methods have advantages and limitations. For example, the electrochemical methods use no chemicals and are energy-efficient [7]. However, electrode fouling, high electrode cost, and low conduction of the polluted water limit their application in water treatment [8]. Fenton and photo-Fenton-like processes are used in degrading antibiotics in wastewater. However, the high cost and excess sludge generation cause additional environmental issues [9,10]. Therefore, developing alternative methods for the destruction of antibiotics in water is timely [11,12,13]. 

Metal–organic frameworks (MOFs) were synthesized by coordinating metal ions or clusters and organic ligands [14,15]. The MOFs have high porosity, specific surface area, tunable channels, enhanced stability, and adjustable bandgap energies. The MOFs are widely used in bio-sensing [16], drug transport [17], adsorption [18], and catalysis [19,20]. UIO-66 (Zr) is an archetypal Zr-based MOF with good thermal and chemical stability [19,20]. However, it shows limited photocatalytic efficiency due to poor charge separation [21,22]. Several chemical routes are available to improve the photocatalytic performance of UIO-66. The heterojunctions UIO-66/g-C_3_N_4_ [23], UIO-66/TiO_2_ [24], and AgI/UIO-66 [25]) can promote the charge separation and migration of photo carriers. Zhou et al. [26] used Ag_2_CO_3_/UIO-66 composite to degrade RhB under visible light radiation. UIO-66 was also functionalized with -NH_2_ and -OH groups to enhance charge-carrying performance. The NH_2_-UIO-66/HO-UIO-66 composites red-shift under visible light with enhanced charge separation, improving the electron transport from the organic connector to the Zr-O cluster [27]. Li et al. [28] hydroxylated UIO-66 with a solvothermal method, viz. (OH)_2_-UIO-66. The (OH)_2_-UIO-66 has stronger visible light absorption than UIO-66; due to the reduction in the bandgap, the surface hydroxyls can act as intra-molecular hole scavengers to facilitate photo-induced charge carrier separation. However, in all these cases, the UIO-66 composites tend to agglomerate, and their catalytic performance is reduced upon repeated use. Carriers such as natural diatomite are also introduced to disperse UIO-66 catalysts to minimize aggregation [29,30,31]. 

In this research, we used hydroxylated UIO-66 on a diatomite carrier (hereafter HO-UIO-66/DE) as a photocatalyst, using a one-step solvothermal synthesis. The presence of hydroxyl sites enhances the catalytic activity. The stability of the HO-UO-66/DE was enhanced by calcination at different temperatures between 100 to 500 °C. SMX was used as an index compound to evaluate the catalytic activity of the materials using a Fenton-like reaction. Electron paramagnetic resonance spectroscopic methods were also used to examine the SMX degradation mechanism by the photo-Fenton HO-UIO-66/DE-300 catalyst. 

## 2. Experiments

### 2.1. Materials and Chemicals

Diatomite was purchased from International Building Materials Environmental Technology Co., Ltd. (Hefei, China). 2, 5-dihydroxyterephthalic acid, zirconium chloride (ZrCl_4_), sodium chloride (NaCl), anhydrous sodium carbonate (Na_2_CO_3_), sodium nitrate (NaNO_3_), and sodium sulfate (Na_2_SO_4_) were purchased from the Sinopharm Chemical Reagent Co., Ltd. (Sinopharm, Shanghai, China). Sulfamethoxazole (SMX), N, N dimethylformamide (DMF), methanol (MeOH), hydrochloric acid (HCl), sodium hydroxide (NaOH), 30% hydrogen peroxide (H_2_O_2_), ammonium acetate (CH_3_COONH_4_), tert-butanol (TBA), ethylenediaminetetraacetic acid disodium salt (EDTA-2Na), 1,4-benzoquinone (BQ), furfuryl alcohol (FFA), and 2,2,6,6-tetramethyl-4-piperidinyl (TEMP) were purchased from the Shanghai Macleans Chemical Co., Ltd., (Sinopharm, Shanghai, China). 5,5-dimethyl-1-pyrroline N-oxide (C_6_H_11_NO, DMPO) was purchased from Sigma-Aldrich (Alfa Aesar, Waltham, MA, USA). All of the chemicals used in the experiment are analytical grade. All of the preparations were carried out using deionized water. 

### 2.2. Synthesis of HO-UIO-66/DE

The HO-UIO-66/DE composites were synthesized via a one-step solvothermal method (Figure S1). First, 1.0 g of diatomite powder was ultrasonicated in 80 mL of DMF solution for 30 min; 233 mg of ZrCl_4_ was added to the diatomite suspension and stirred for 1 h, then 396 mg 2, 5-dihydroxy terephthalic acid was added. Finally, the mixed solution was transferred to an autoclavable polytetrafluoroethylene vessel and kept at 120 °C for 24 h. The substrate was washed with DMF and methanol to remove impurities. The solid–solution separation was achieved by centrifugation. The cleaned, powdered catalyst was stored at 60 °C in an oven for 8 h. The resulting catalytic materials were calcinated at 300, 400, and 500 °C for 2 h using 5 °C/min heating rates. The products were named as HO-UIO-66/DE-0 (no calcination), HO-UIO-66/DE-300 (300 °C calcination), HO-UIO-66/DE-400 (400 °C calcination), and HO-UIO-66/DE-500 (500 °C calcination). 

The UIO-66/DE-0 and UIO-66/DE-300 were synthesized as described above, replacing 396 mg of 2, 5-dihydroxy terephthalic acid ligand with 332 mg of terephthalic acid. The other preparations were synthesized as described above.

### 2.3. Catalyst Characterization

The morphology, microstructure, and elemental distribution of the samples were investigated using cold field emission scanning electron microscopy (FESEM, SU-8020 Hitachi, Tokyo, Japan), transmission electron microscopy (TEM, JEM-2100F HRTEMJEOL, Tokyo, Japan), and energy dispersive X-ray diffraction spectroscopy (Ultim Extreme, Oxford, UK). The crystal structure of the samples was analyzed in the range of 2θ 5–80° using an X-Pert fixed-target X-ray diffractometer (PRO MPD XRD, PANalytical, Almelo, The Netherlands). The surface functional groups of the samples were examined using Fourier transform infrared spectroscopy (FT-IR, Thermo Nicolet, MA, USA). The surface properties of the samples were studied with X-ray photoelectron spectroscopy (XPS, Thermo, MA, USA). UV–vis-NIR with the diffuse reflectance sampling holder (DRS) was used to examine the light absorption via a UV–Vis-NIR spectrophotometer (CARY 500 UV-Vis-NIR, Santa Clara, CA, USA). The photoelectrochemical measurements are detailed in S1 (support documentation).

### 2.4. Photo-Fenton Catalytic Activity

The effect of calcination temperatures of the catalysts on the photocatalytic performance was determined using a 500 W xenon lamp as a light source and SMX as an index pollutant. In the catalytic degradation experiment, 400 mg/L catalyst was added to a 20 mg/L SMX aqueous solution (50 mL). The pH value of the solution was adjusted to the desired value using ~0.1 M HCl or ~0.1 M NaOH. After the catalyst was added to the solution, the dark reaction lasted for 30 minutes to reach the adsorption and desorption balance. Then, the reaction mixture was irradiated by a 500 W Xe lamp, and an aliquot of the suspensions was taken at 30-minute intervals. The aliquots were filtered through a 0.45 nm membrane to remove the particulate matter.

## 3. Analysis Methods

The SMX and degradation products were analyzed via reversed-phase high-performance liquid chromatography (RP-HPLC, Shimadzu LC-20A, Kyoto, Japan), using a UV–Vis detector (SPD-20A, Shimadzu, Kyoto, Japan) and a C18 column (4.6 × 250 mm) at λ 255 nm with a 40% acetonitrile and 60% 10 mM ammonium acetate mobile phase at a 1.0 mL/min flow rate. The total organic carbon (TOC) in the solution was determined with a TOC/TN analyzer (Multi N/C 3100, Analytik Jena AG, Thuringia, Germany), and the pH measurements were made using a pH meter (HACHHQ30d, HACH, CO, USA). The electron paramagnetic resonance spectroscopic method (ESR, Bruker, Germany) was used to detect •OH, •O_2_^−^, and ^1^O_2_ radicals, using DMPO and TEMP as scavengers. The intermediates were analyzed with high-performance liquid chromatography-mass spectrometry, using the same column and solvent system used in SMX quantification (RP-HPLC-MS; Agilent 1290/6460, Santa Clara, CA, USA).

## 4. Results and Discussion

### 4.1. Structural and Morphological Characterizations

**Figure 1:**
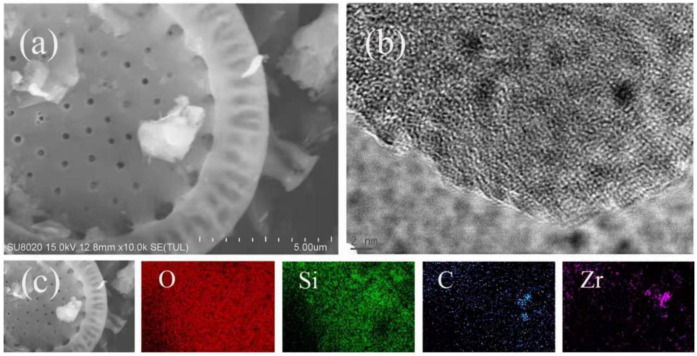
SEM (**a**) and HRTEM images (**b**) and elemental mapping images (**c**) of HO-UIO-66/DE-300.

Micrographic structures and morphologies of the HO-UIO-66/DE-300 composite were examined using FESEM and HRTEM spectroscopic methods, as shown in Figure 1a–c. Calcination of the HO-UIO-66/DE at 300 °C shows optimal photocatalytic activity for SMX degradation. The calcination temperature of the substrate shows a marked effect on the catalytic activity of the HO-UIO-66/DE composites, and both the micrographic structure and morphology depend on it. As shown in the FESEM data (Figure 1a), the grain structure of our composites is similar to the well-known UIO-66 metal–organic framework [32]. The HO-UIO-66 particulates were uniformly grown on the diatomite surface (Figure 1a). The average grain width of the particulates is about 400 nm. No lattice fringes were observed on the surface of HO-UIO-66 (Figure 1b). Figure 1c is the EDS mapping data of the HO-UIO-66/DE-300 sample. As shown in the data, the C, O, and Zr elements in the HO-UIO-66/DE-300 catalyst are evenly distributed on the surface of the diatomite, and the MOF was successfully synthesized on diatomite.

The XRD data of all of the samples are shown in Figure S1. The broad diffraction peak at 22.1° is typical of the amorphous nature of diatomite, and the peak at 26.6° corresponds to the (101) plane of the SiO_2_ structure [30] (JCPDS No. 46-1045). The characteristic peak at 7.5° corresponds to the (111) crystal plane of HO-UIO-66 [33]. Due to the low content of HO-UIO-66, the other peaks are weak or not readily discerned. Upon calcination, the XRD peak at (111) also decreased, showing that the crystal structure of the HO-UIO-66 diminished upon calcination. The XRD data of HO-UIO-66 and HO-UIO-66/300 are shown in Figure S3. The characteristic peak at 7.5° corresponds to the (111) crystal plane, and the XRD peak at (111) also decreased after calcination.

**Figure 2:**
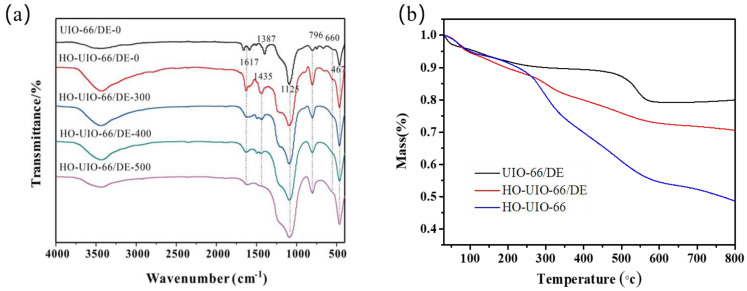
The FT−IR spectra (**a**) and TG analyses (**b**) of synthesized photocatalysts.

The FT-IR spectra of catalyst samples synthesized at different calcination temperatures are shown in Figure 2a. Compared with the UIO-66/DE-0, the HO-UIO-66/DE-0 peak has obvious hydroxyl groups on the surface of the catalyst. The peak between 3700 cm^−1^–3000 cm^−1^ and the peak at 1617 cm^−1^ are attributed to the stretching vibrations of hydroxyl functional groups in the sample [28]. With the increasing calcination temperature, the vibrational tensile peak of the hydroxyl group decreases. The IR bands at 1435 cm^−1^ and 1387 cm^−1^ correspond to the -COOH functional group in the ligand [34]. With the increase in the calcination temperature, this band intensity decreases, which implies a reduction in surface defect sites [28]. The 660 cm^−1^ is ascribed to Zr-O bond vibrations [35]. For diatomite, the sharp band at 796 cm^−1^ was observed at strong asymmetric Si-O-Al stretching vibrations, and the bands at 1125 cm^−1^, 796 cm^−1^, and 467 cm^−1^ correspond to the structural deformation of diatomite [36,37].

The thermal stability of the UIO-66/DE-0, HO-UIO-66/DE-0, and HO-UIO-66 catalysts was determined under a nitrogen atmosphere using thermogravimetric analysis (TGA), and the results are shown in Figure 2b. The UIO-66/DE-0 catalyst showed higher stability. The hydroxyl group in the HO-UIO-66/DE-0 and HO-UIO-66 catalysts suffers serious weight loss with the increase in temperature. According to previous research, weight loss was allocated to the following processes. The weight loss of the three catalysts before 100 °C is attributed to the volatilization of the solvent [38]. The removal of 2, 5-dihydroxyterephthalate ligands and the dihydroxylation of Zr6 clusters occur between 100 °C to 350 °C temperature range [39]. Within this range, the density of -COOH is decreased, while the catalytic activity shows a maximum decline at 350 °C [22]. Therefore, the weight loss of the HO-UIO-66/DE-0 and HO-UIO-66 catalysts is more serious in this temperature range. The observed weight loss between 350 °C to 500 °C is due to the decomposition of the structural framework of the catalyst [39]. 

To investigate the chemical composition of the catalysts at different calcination temperatures, XRF analyses were carried out on HO-UIO-66/DE-300, 400, and 500, and the data are shown in Table S1. The results show that the relative contents of Si and Zr in the catalysts increased after calcination, and the relative contents of C, N, and O decreased, which may be due to the decomposition of water and organic matter in the catalysts with the increase in calcination temperature.

**Figure 3:**
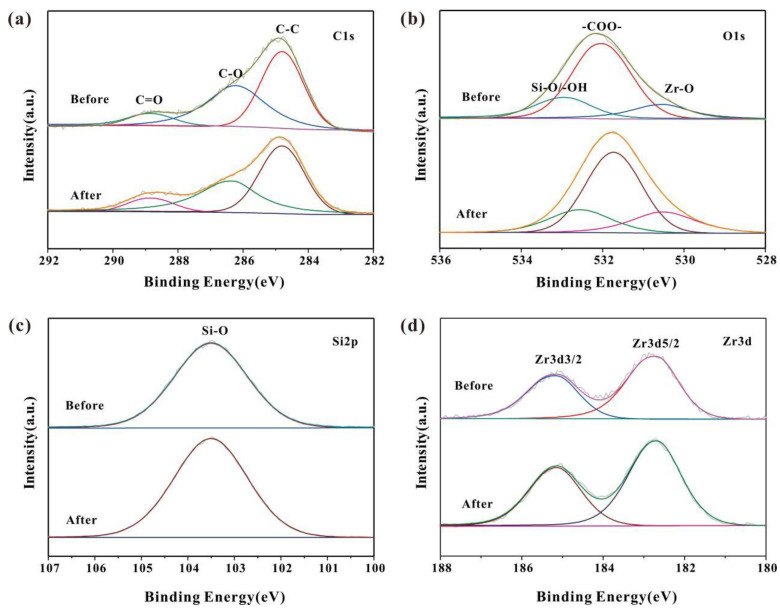
XPS spectra of C1s (**a**), O1s (**b**), Si2p (**c**), and Zr3d (**d**) for HO-UIO-66/DE-300.

The near-surface chemical composition of the HO-UIO-66/DE-300 catalyst before and after calcination was examined using XPS. The C1s and O1s peaks of the catalysts show similar trends before and after calcination. The C1s peak was decomposed into three components at 284.8 eV (C-C bond), 286.2 eV (C-O bond), and 288.8 eV (C=O); these functional groups correspond to the presence of 2, 5-dihydroxyterephthalic acid(Figure 3a) [28]. Upon calcination, the intensity of the C-O peak decreased from 31.5% to 27.7%, whereas the C=O bond intensity increased from 8.93% to 12.0%. The deconvoluted components of the O1s peak are ascribed to Zr-O at 530.5 eV, -COO- at 532.0 eV [40], and H-O/Si-O at 532.9 eV(Figure 3b) [28]. Upon interactions of HO-UIO-66/DE with SMX, the peak at 532.0 eV shows a slight shift. The shift of the C=O bond is due to SMX adsorption on the catalyst. As shown in Figure 3c, the peaks at 103.5 eV, 182.7 eV, and 185.1 eV are due to Si-O, Zr3d5/2, and Zr3d3/2, respectively. The Si-O peak resulted from diatomite. The XPS peaks of Zr3d5/2 and Zr3d3/2 did not show any variations upon calcination(Figure 3d). The XPS spectral signatures confirm the formation of HO-UIO-66/DE-300.

**Figure 4:**
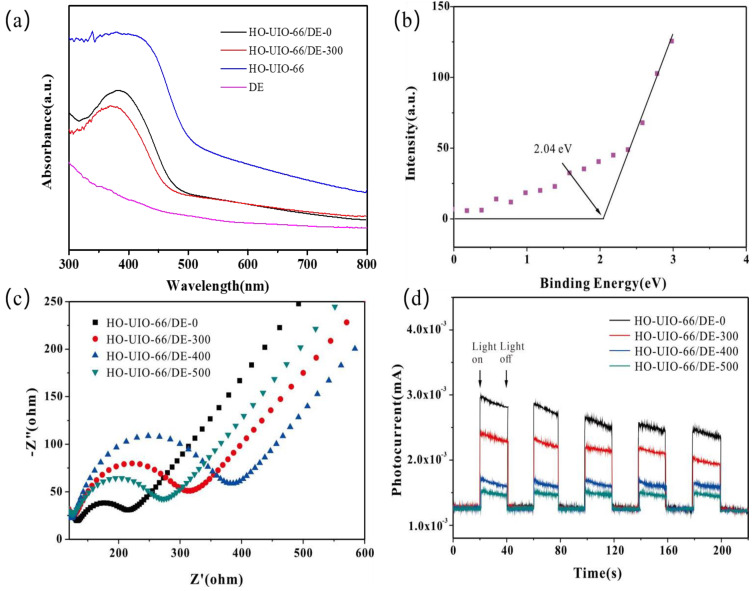
UV−DRS spectra (**a**), VB XPS spectrum of HO-UIO-66/DE-300 (**b**), EIS Nyquist plots (**c**), and (**d**) photocurrent responses of synthesized photocatalysts.

UV–Vis diffused reflectance spectroscopy (DRS) was used to evaluate the effect of calcination temperature on the optical properties and bandgap energy of the DE, HO-UIO-66, and HO-UIO-66/DE composites. As shown in Figure 4a, HO-UIO-66, HO-UIO-66/DE-0, and HO-UIO-66/DE-300 show an absorption edge around λ = 500 nm. The absorption spectrum of the HO-UIO-66/DE-300 composite shows a blue shift referenced to HO-UIO-66/DE-0, showing structural alterations of the catalyst upon calcination. Furthermore, the bandgap energy (Eg) was estimated via a Tauc plot using the Tauc equation:

αhv = A(hv–E_g_)^n/2^ (1)

where α, A, h, and v stand for the absorption coefficient, the proportionality constant, Planck’s constant, and light frequency, respectively. The parameter *n* = 1 for UiO-66 [24]. The relationship between (ahv)^1/2^ and photon energy (hv) is shown in Figure S4a. The calculated band gap increases from 2.61 eV for HO-UIO-66/DE-0 to 2.68 eV for HO-UIO-66/DE-300. Figure 4b is the valence band XPS of HO-UIO-66/DE-300. The calculated valence and conduction band potentials of HO-UIO-66/DE-300 are 2.04 eV and −0.64 eV, respectively.

The electrochemical impedance spectroscopic (EIS) measurements were carried out to probe the electron transfer between the electrode surface and the solution interface. As shown in Figure 4c, the diameter of the Nyquist arc of the HO-UIO-66/DE-0 catalyst is the smallest. With the increase in calcination temperature, the diameter of the Nyquist arc has increased, reaching an optimal value at 400 °C. When the calcination of the substrate was performed at 500 °C, the electron transfer rate increased due to ligand carbonization. The Tafel polarization plots were constructed using the materials received under calcination at different temperatures in Figure S4b. As the calcination temperature increases, the catalyst’s corrosion current increases steadily as a result of the enhanced stability of the material. Although HO-UIO-66/DE-0 shows the highest efficiency as a catalyst, it is not stable. However, HO-UIO-66/DE-0 shows comparable catalytic efficiency for SMX degradation with enhanced stability. Hence, this material was used in our other experiments. 

Figure 4d shows the photocurrent responses of the catalysts fabricated at different calcination temperatures. A xenon lamp was used to irradiate the catalyst. The HO-UIO-66/DE-0 catalyst shows the highest photocurrent response under irradiation due to suppressing electron–hole recombination. In agreement with the EIS data, the photocurrent response of the calcined catalysts is not efficient. 

### 4.2. Photo-Fenton Catalytic Activity Analysis

**Figure 5:**
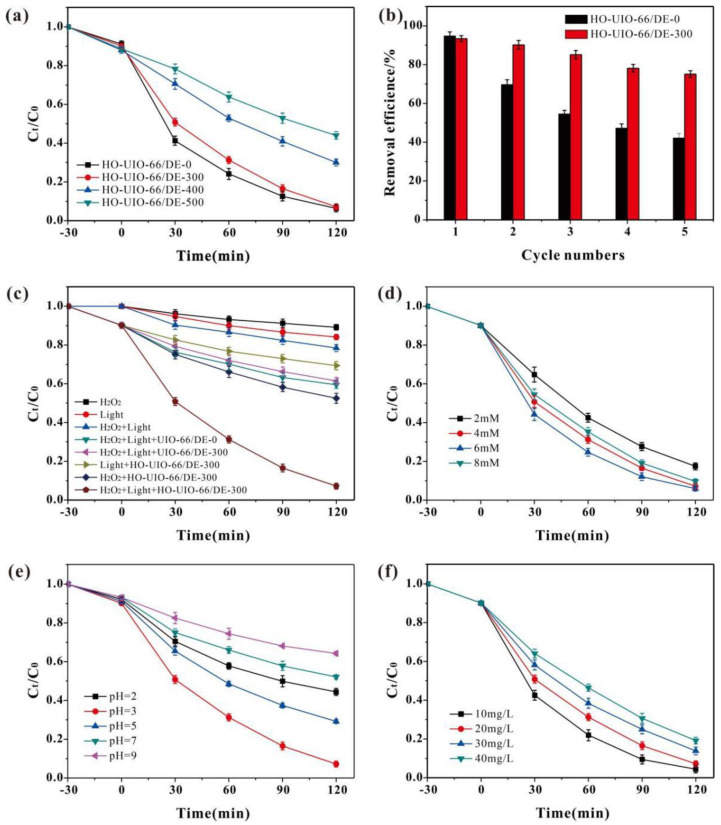
The effect of different catalysts’ degradation of SMX in photo−Fenton system (**a**). The removal efficiency of SMX by two catalysts after 5 cycles of reaction (**b**). The effect of different reaction systems’ degradation of SMX in photo−Fenton system (**c**). The effect of H_2_O_2_ concentration of SMX in photo−Fenton system (**d**). The effect of pH of SMX in photo−Fenton system (**e**). The effect of initial SMX concentration of SMX in photo−Fenton system (**f**) (reaction conditions: pH 3.0; initial SMX, 20.0 mg/L; catalyst, 400 mg/L; H_2_O_2_, 4.0 mM).

The efficiency of SMX degradation by the four photo-Fenton catalysts was measured as a function of the UIO-66/DE calcination temperature, initial concentration of H_2_O_2_, solution pH, and SMX loading. As shown in Figure S5, the SMX adsorption by the catalysts became optimal after 30 min. After this time, the SMX-sorbed catalysts were irradiated using visible light for 120 min. The SMX degradation results are shown in Figure 5a. The SMX degradation efficiencies of HO-UIO-66/DE-0, HO-UIO-66/DE-300, HO-UIO-66/DE-400, and HO-UIO-66/DE-500 are 94.7%, 93.8%, 69.9% and 56.0%, respectively (HO hydroxylation, DE diatomite). When the calcination temperature increases, the SMX degradation efficiency by the substrates varied, showing an optimal value at 300 °C calcination. With a further increase in the calcination temperature, the hydroxyl groups on the catalyst seem destroyed. 

The stability of the HO-UIO-66/DE-0 and HO-UIO-66/DE-300 catalysts was further studied. For these experiments, 0.40 g of catalyst, 20 mg/L SMX, and 4 mM H_2_O_2_ were used in a pH 3 solution. After each cycle, the used catalyst was washed three times with deionized water and collected using centrifugation. The material was dried at 80 °C to commence the next cycle. This process was repeated five times. The efficiency of SMX degradation is shown in Figure 5b. The degradation efficiency of the HO-UIO-66/DE-300 catalyst for SMX decreased from 93.8% to 75.1% at the end of the fifth cycle, while the degradation efficiency of the HO-UIO-66/DE-0 catalyst for SMX decreased from 94.7% to 42.1%. Therefore, the calcined HO-UIO-66/DE-300 catalyst has a more stable structure and catalytic performance. Therefore, HO-UIO-66/DE-300 was used in subsequent experiments.

The effects of visible light irradiation and H_2_O_2_ concentration on the catalytic efficiency of HO-UIO-66/DE-300 were also examined, as shown in Figure 5c. In the absence of the catalyst, visible light irradiation and H_2_O_2_ concentration exert no significant effect on SMX degradation. When H_2_O_2_ and visible light were added, the degradation rate of the UIO-66/DE-0 and UIO-66/DE-300 catalysts on SMX was not obvious. Under dark conditions, the Fenton-like reaction was carried out by adding H_2_O_2_ and HO-UIO-66/DE-300, and the degradation efficiency of SMX was only 44.6%. When irradiated with visible light, the SMX degradation efficiency was low. Under the cooperative condition of visible light and H_2_O_2_, many reactive oxygen species (ROS) were produced in the HO-UIO-66/DE-300/light/H_2_O_2_ system, and the degradation efficiency of SMX was significantly improved [41]. 

Figure 5d shows the effect of H_2_O_2_ concentration on the degradation of SMX by HO-UIO-66/DE-300. When the H_2_O_2_ concentration increased from 2 mM to 6 mM, the degradation rate increased significantly. However, when H_2_O_2_ concentration was beyond 6 mM, the degradation effect of the catalyst decreased, which may be due to the excessive H_2_O_2_ concentration inhibiting ROS production in the system. According to our results, when the H_2_O_2_ concentration is beyond 4 mM, the final degradation of SMX is not obvious. From an economic point of view, the removal of SMX is optimal at a concentration of 4 mM H_2_O_2_.

The solution pH plays an important role in the HO-UIO-66/DE-300/light/H_2_O_2_ system [42,43]. As shown in Figure 5e, the SMX degradation efficiency by HO-UIO-66/DE-300 depends on the solution pH. When the solution pH was increased from 2 to 3, the degradation efficiency of SMX increased by 37.2% within 120 min. Under extremely acidic conditions, •OH radicals can be scavenged by H^+^ [44,45], and the corrosion rate of the catalyst increases, affecting the stability of the catalyst, which inhibits SMX degradation under extremely acidic conditions. As the pH increased from 3 to 9, the degradation efficiency of SMX decreased from 93.8% to 35.7%. When the pH is increased, the SMX degradation efficiency decreases because the redox potential of ROS decreases, favoring H_2_O_2_ decomposition [42,46]. Therefore, HO-UIO-66/DE-300 had the highest degradation SMX efficiency at pH 3.

The effect of different calcination temperatures on the photocatalytic degradation efficiency of catalysts was evaluated by changing the initial SMX concentration, and the results are shown in Figure 5f. When the initial concentration of SMX is low, the degradation efficiency is obvious. With the increase in the initial concentration of SMX, the availability of the active site on the catalyst is limited, and the degradation efficiency decreases.

**Figure 6:**
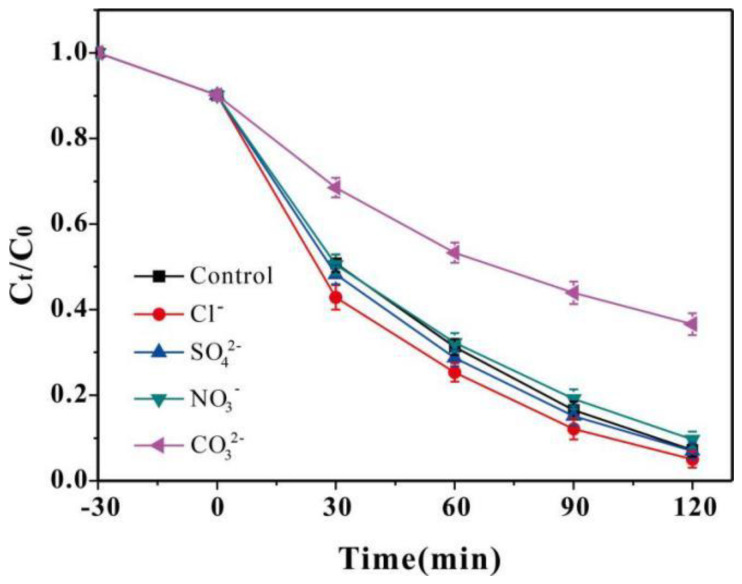
Effects of various anions on SMX degradation (reaction conditions: pH 3.0; initial SMX, 20.0 mg/L; catalyst, 400 mg/L; H_2_O_2_, 4.0 mM; anion concentration, 10 mM).

Under the optimal conditions used, the effects of Cl^−^, SO_4_^2−^, NO_3_^−^, and CO_3_^2−^ on SMX degradation were examined, as shown in Figure 6. In agreement with previous data [47], the degradation of SMX accelerated after the addition of Cl^−^. The Cl^−^ is an effective •OH scavenger; however, by this process, Cl• can be generated, which favors the rapid degradation of SMX due to the relatively high standard redox potential of Cl• being similar to that of •OH [48,49,50]. The presence of NO_3_^−^ and SO_4_^2−^ had no significant effect on the degradation of SMX. In addition, with the addition of CO_3_^2−^, the degradation efficiency of SMX decreased due to the quenching of •OH and •O_2_^−^ [51].

### 4.3. SMX Degradation in Actual Water

To further investigate the actual performance of the HO-UIO-66/DE-300/light/H_2_O_2_ system, we chose medical wastewater as the substrate. The medical wastewater was taken from the conditioning tank of the wastewater treatment plant of the Affiliated Hospital of Hefei University of Technology. The characteristics and compositions of the water samples are shown in Table S1. The concentration of SMX added to the collected medical wastewater was 8.5 mg/L. The concentration of SMX added to the collected medical wastewater was 8.5 mg/L. The degradation rates of SMX contaminants in deionized water and medical wastewater were 80% and 73%, respectively, within 120 min (Figure S10). The lower degradation rate of SMX in medical wastewater compared to deionized water was attributed to free radical competition between SMX and natural organic matter present in the water. Overall, the HO-UIO-66/DE-300/light/H_2_O_2_ system showed good SMX removal efficiency in real water.

## 5. Analysis of SMX Degradation Possible Pathway 

**Figure 7:**
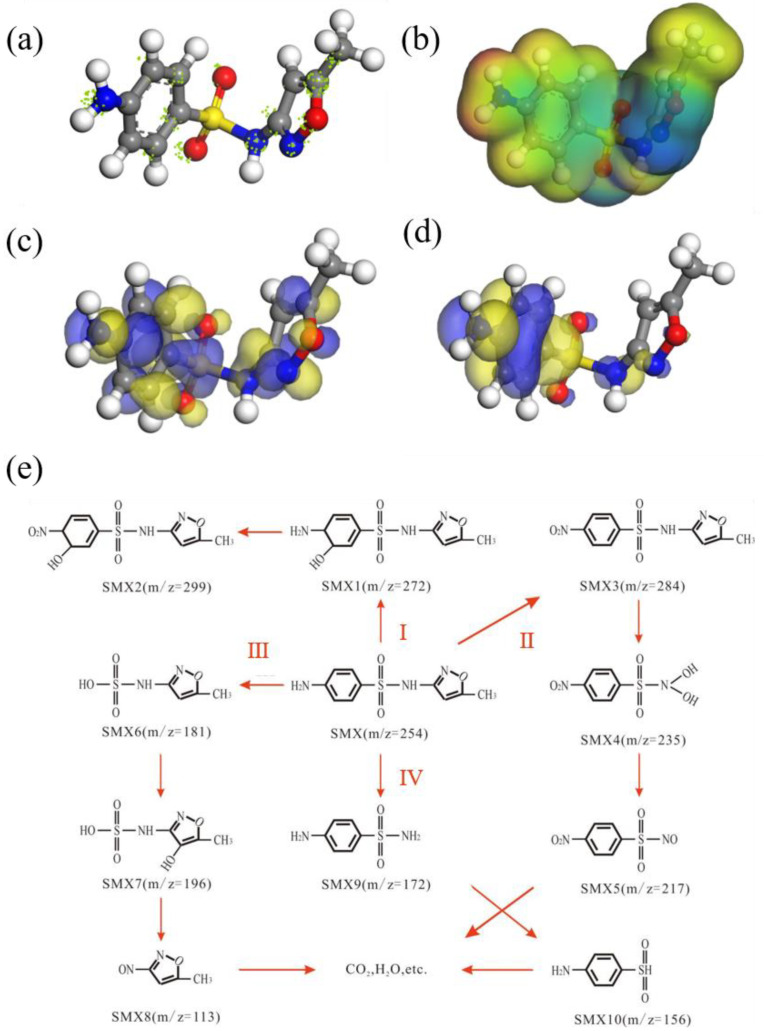
(**a**) Calculation of reactive sites on SMX via Fukui function; (**b**) electron density distribution of SMX, where red represents regions with high electron density and blue represents regions with low electron density; LUMO orbital energy (**c**) and HOMO orbital energy (**d**) of SMX; (**e**) proposed degradation pathways of SMX in the HO-UIO-66/DE-300 photo-Fenton system.

As shown in Figure S6, after 5 cycles, approximately 51.1% of the TOC of SMX solution was degraded, which means that SMX can be mineralized into smaller molecules in the HO-UIO-66/DE-300/H_2_O_2_/light system. Five possible degradation pathways were proposed based on the degradation of SMX and the detected intermediates (Figure 7e and Table S2). The mass spectra of the SMX intermediates are shown in Figure S7, as well as in combination with the calculation of the reactive sites on SMX via the Fukui function (Figure 7a) and the electron density distribution of SMX (Figure 7b). In pathway I, the benzene ring on SMX (*m*/*z* = 254) with high HOMO orbital energy is readily broken and attacked by electrophilic hydroxyl groups and adducted to form SMX1, followed by oxidation of amino (-NH_2_) to nitryl (-NO_2_) of SMX1 (*m*/*z* = 272) to form SMX2 (*m*/*z* = 299) (Figure 7c,d) [52]. In pathway II, the amino group (-NH_2_) on the benzene ring of SMX is directly oxidized to a nitro group (-NO_2_) to form SMX3 (*m*/*z* = 284). Subsequently, the sulfur–nitrogen bond in SMX3 is directly cleaved. At the same time, it combines with the hydroxyl group in solution to generate SMX4 (*m*/*z* = 235), which forms SMX5 (*m*/*z* = 217) due to the attack of the superoxide radical with electrophilic nature on the -NH_2_ group. The nitro derivatives undergo a series of oxidation reactions to generate small molecules [53]. In pathway III, the S-C bond of SMX is attacked by hydroxyl radicals and superoxide radicals to break SMX6 (*m*/*z* = 181), then undergoes hydroxyl addition to form SMX7 (*m*/*z* = 196), which further oxidizes the amino group (-NH_2_) to the nitro group (-NO_2_) to form SMX8 (*m*/*z* = 113), and then undergoes an oxidation reaction to generate small molecular species [54,55]. In pathway IV, the C-N bond of high HOMO is oxidized and broken to form SMX9 (*m*/*z* = 172), and then the S-N bond in SMX9 is oxidized and broken to form SMX10 (*m*/*z* = 156), which is further oxidized to form small molecules [56].

## 6. Degradation Mechanism of HO-UIO-66/DE-300 in Photo-Fenton System

**Figure 8:**
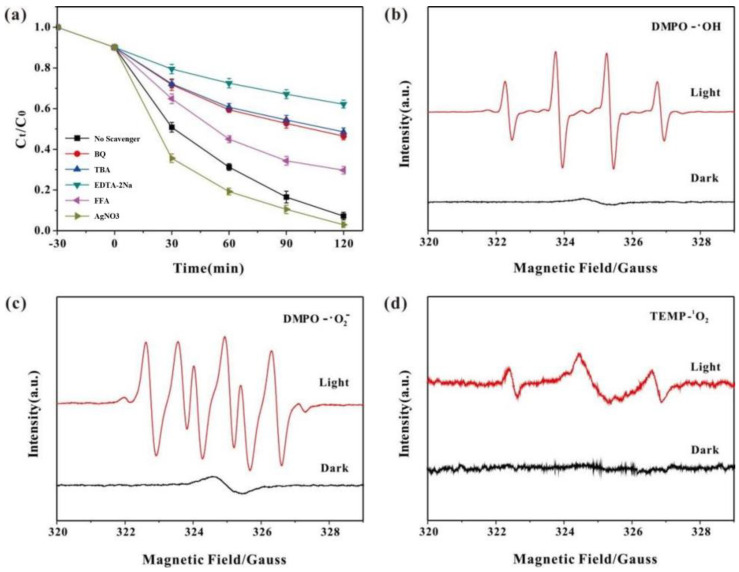
(**a**) Effect of radical scavengers on the degradation of SMX (reaction conditions: pH 3.0; initial SMX, 20.0 mg/L; catalyst, 400 mg/L; H_2_O_2_, 4.0 mM); ESR spectra of •OH (**b**), •O_2_^−^ (**c**), and ^1^O_2_ (**d**) generated by the catalyst in the presence and absence of visible light irradiation.

To examine the degradation mechanism of SMX by HO-UIO-66/DE-300 in the photo-Fenton system, we also studied the effect of h^+^, •OH, •O_2_^−^, and ^1^O_2_ radicals by scavenging them with quenchers. To identify the active species in the HO-UIO-66/DE-300/light/H_2_O_2_ system, we used the following radical quenchers: tert-butanol (TBA, 100 mM), p-benzoquinone (BQ, 4 mM), EDTA-2Na (4 mM), AgNO_3_ (10 mM), and FFA (10 mM). TBA is an effective quencher that inhibits the formation of •OH in the reaction system, BQ can be used as a trap for •O_2_^−^ formed during the reaction, and EDTA-2Na can scavenge holes generated by the UIO-66/DE-300 catalyst. The AgNO_3_ can scavenge the electrons generated by the HO-UIO-66/DE-300 catalyst, and FFA can inhibit the formation of ^1^O_2_ in the reaction system. As shown in Figure 8a, after adding excess AgNO_3_, the SMX degradation rate increased after 120 min, which may be due to the formation of heterojunction with the catalyst after the addition of AgNO_3_, inhibiting the recombination of electrons and holes, indicating that the removal of e- does not affect the progress of the reaction system. After the addition of excessive EDTA-2Na, BQ, TBA, and FFA, the degradation efficiency of SMX decreased significantly, from 93.8% to 37.8%, 53.6%, 51.5%, and 70.3%, respectively. Therefore, through experiments, we found that h^+^, •OH, •O_2_^−^, and ^1^O_2_ play an important role in SMX degradation, among which h^+^ plays a particularly significant role.

The ESR spectroscopic method was used to examine the production of •OH, •O_2_^−^, and ^1^O_2_ by the HO-UIO-66/DE-300 photo-Fenton system. As shown in Figure 8b–d, under darkness, no •OH, •O_2_^−^, or ^1^O_2_ ESR signals are observed, viz. no DMPO-•OH, DMPO-•O_2_^−^, or TEMP-^1^O_2_ ESR signals are shown. When the reactor is irradiation with a 500 W xenon lamp, •OH, •O_2_^−^, and ^1^O_2_ quencher signals, viz., DMPO-•OH, DMPO-•O_2_^−^, or TEMP-^1^O_2_ are readily observed. The •OH, •O_2_^−^, and ^1^O_2_ radicals present in the HO-UIO-66/DE-300 photo-Fenton reactor play an important role in SMX degradation, consistent with the results obtained for free radical scavenging experiments.

**Figure 9:**
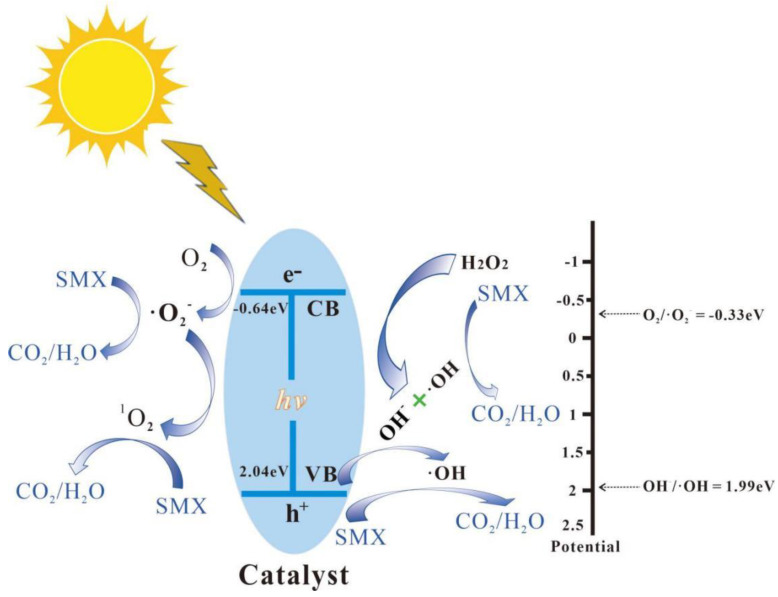
Possible photo-Fenton degradation mechanism.

As shown in Figure 9, we proposed a possible mechanism of SMX degradation by HO-UIO-66/DE-300 under light irradiation. Electrons and holes are photo-generated upon visible light irradiation of HO-UIO-66/DE-300, enhancing a charge transfer between ligand and metal clusters. Due to the negative CB potential (−0.64 eV) of HO-UIO-66/DE-300, the excited electrons can reduce the dissolved O_2_ in solution to form •O_2_^−^ [40], and it can combine with the h^+^ generated by HO-UIO-66/DE-300 to generate ^1^O_2_, and the generated e^−^ reacts with H_2_O_2_ to generate •OH and OH^−^; OH^−^ is continuously oxidized to •OH by h^+^ [57]. 

Part of the reaction process can be described as follows (Equations (2)–(7)):

Catalyst + *hv* → h^+^ + e^−^ (2)

H_2_O_2_ + e^−^ → •OH + OH^−^ (3)

OH^−^ + h^+^ → •OH + e^−^ (4)

h^+^+ •O_2_^−^ → ^1^O_2_ (5)

e^−^ + O_2_ → •O_2_^−^ (6)

SMX + h^+^/•OH /•O_2_^−^ /^1^O_2_ →produces (7)

Therefore, h^+^, •OH, •O_2_^−^, and ^1^O_2_ all play an important role in the degradation of SMX in the HO-UIO-66/DE-300/light/H_2_O_2_ system.

## 7. Conclusions

A novel diatomite-supported hydroxyl-modified UIO-66 catalyst (HO-UIO-66/DE-300) was synthesized using a solvothermal method. In degrading SMX, the stability of the HO-UIO-66/DE-300 was enhanced by calcination at 300 °C. The SMX degradation by HO-UIO-66/DE-300 is optimal with 4 mM [H_2_O_2_] and pH 3. The HO-UIO-66/DE-300-assisted SMX degradation occurs via a free radical pathway incorporating h^+^, •OH, •O_2_^−^, and ^1^O_2_. The HO-UIO-66/DE-300 has good stability after repeated use. These results indicate that the HO-UIO-66/DE-300 photo-Fenton catalyst has great potential in degrading recalcitrant organic pollutants in water.

## Data Availability

All data generated or analyzed during this study are included in this published article and its supplementary information files.

## Supplementary Materials

The following supporting information can be downloaded at: https://www.mdpi.com/article/10.3390/nano13243116/s1, Figure S1: Schematic of the synthesis process of HO-UIO-66/DE-300; Figure S2: The XRD patterns of synthesized photocatalysts; Figure S3: The XRD patterns of HO-UIO-66 and HO-UIO-66/300; Figure S4: (a) Tauc plots of as-prepared catalysts and (b) Tafel polarization curves; Figure S5: The effect of different catalysts on SMX adsorption performance (reaction conditions: initial SMX, 20.0 mg/L; catalyst, 400 mg/L); Figure S6: Removal efficiency of TOC (reaction conditions: pH 3.0; initial SMX, 20.0 mg/L; catalyst, 400 mg/L; H_2_O_2_, 4.0 mM); Figure S7: Liquid-mass diagram of SMX and its intermediates in photo-Fenton system; Table S1: The chemical properties of HO-UIO-66/DE-0, HO-UIO-66/DE-300, HO-UIO-66/DE-400, and HO-UIO-66/DE-500; Table S2: The structural information of the possible intermediate products.
