# Supplementary material for: Efficient Degradation of Sulfamethoxazole by Diatomite-Supported Hydroxyl-Modified UIO-66 Photocatalyst after Calcination
Source: Nanomaterials (Basel). 2023 Dec 11;13(24):3116. doi: 10.3390/nano13243116 (PMC10745632; doi:10.3390/nano13243116)
Supplement: Supplementary file 1 [file nanomaterials-13-03116-s001.zip › nanomaterials-2716804-supplementary.pdf]

nanomaterials

Supplementary Materials

Efficient Degradation of Sulfamethoxazole by Diatomite-Supported Hydroxyl-Modified UIO-66 Photocatalyst after Calcination

Hui-Lai Liu [1,2], Yu Zhang [1,2], Xin-Xin Lv [1,2], Min-Shu Cui [1], Kang-Ping Cui [1], Zheng-Liang Dai [3], Bei Wang [3], Rohan Weerasooriya [2,4] and Xing Chen [1,2,4,*]

[1] Key Laboratory of Nanominerals and Pollution Control of Higher Education Institutes, School of Resources and Environmental Engineering, Hefei University of Technology, Hefei 230009, China; 18255110208@163.com (H.-L.L.); 18691965261@163.com (Y.Z.); lvx1996@163.com (X.-X.L.); mcui@mail.ustc.edu.cn (M.-S.C.); cuikangping@hfut.edu.cn (K.-P.C.)

[2] Key Lab of Aerospace Structural Parts Forming Technology and Equipment of Anhui Province, Institute of Industry and Equipment Technology, Hefei University of Technology, Hefei 230009, China; rohan.we@nifs.ac.lk

[3] Anqing Changhong Chemical Co., Ltd., Anqing 246002, China; 18256240803@163.com (Z.-L.D.); 13855694589@163.com (B.W.)

[4] National Centre for Water Quality Research, National Institute of Fundamental Studies, Hantana, Kandy, Sri Lanka; rohan.we@nifs.ac.lk

* Correspondence: xingchen@hfut.edu.cn

Photoelectrochemical measurements

The photocurrent measurements were carried out on a CHI-760E electrochemical workstation (Chenhua Instrument, Shanghai, China) in a conventional three-electrode configuration with the sample modified ATO (20 mm * 50 mm) as the working electrode, Pt as the counter electrode, and Ag/AgCl as the reference electrode. The current measurement i–t curve was performed using an LED lamp (35 W) at 0.4 V bias voltage potential. An aqueous solution of 0.5 M Na_2SO_4 was used as the electrolyte.

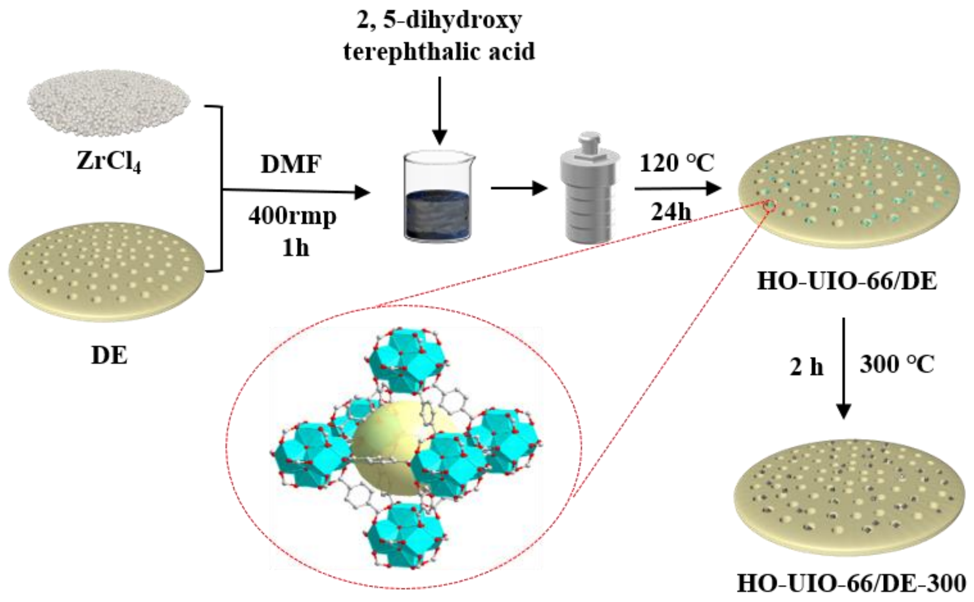

Figure S1. Schematic of the synthesis process of HO-UIO-66/DE-300.

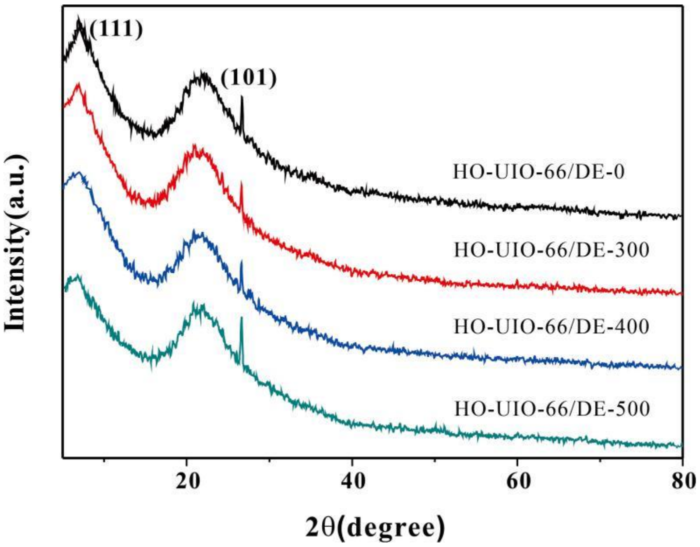

Figure S2. The XRD patterns of synthesized photocatalysts.

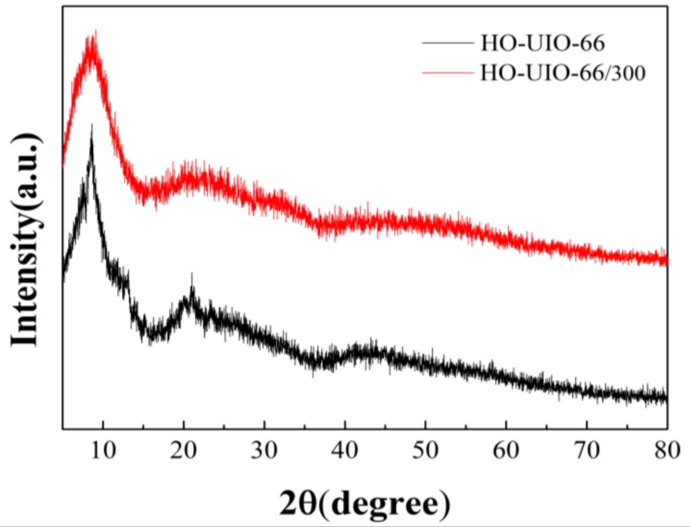

Figure S3. The XRD patterns of HO-UIO-66 and HO-UIO-66/300.

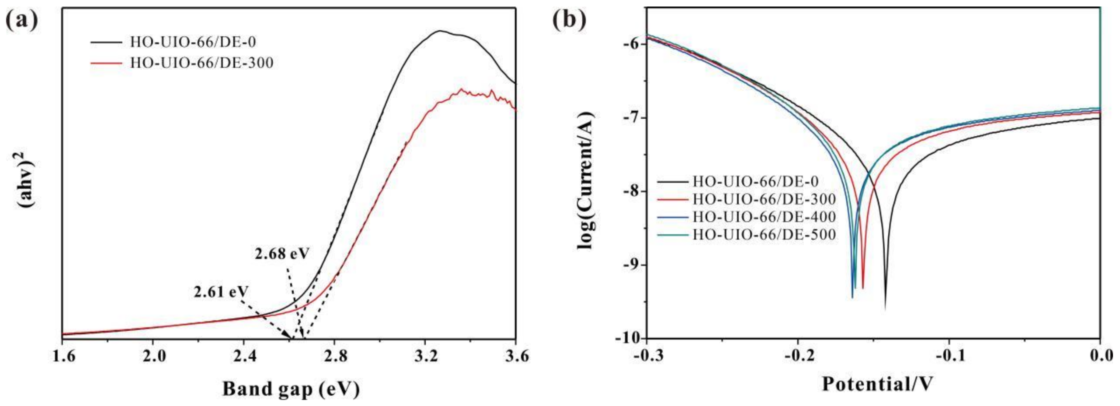

Figure S4. (a) Tauc plots of as-prepared catalysts and (b) Tafel polarization curves.

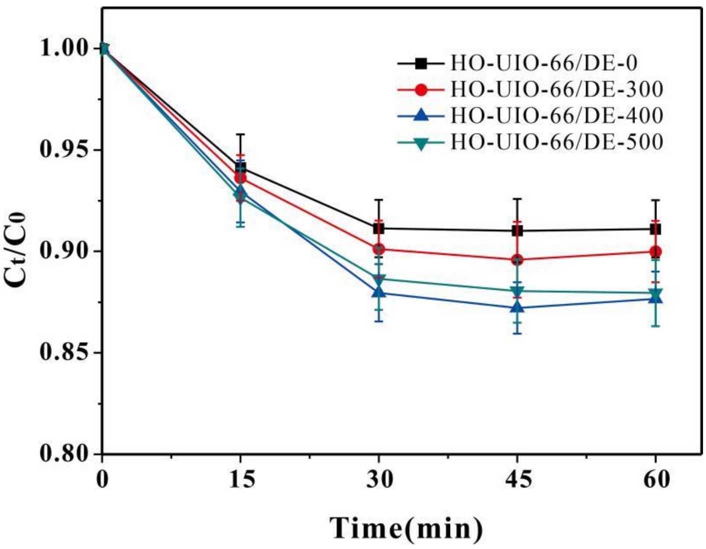

Figure S5. The effect of different catalysts on SMX adsorption performance (reaction conditions: initial SMX, 20.0 mg/L; catalyst, 400 mg/L).

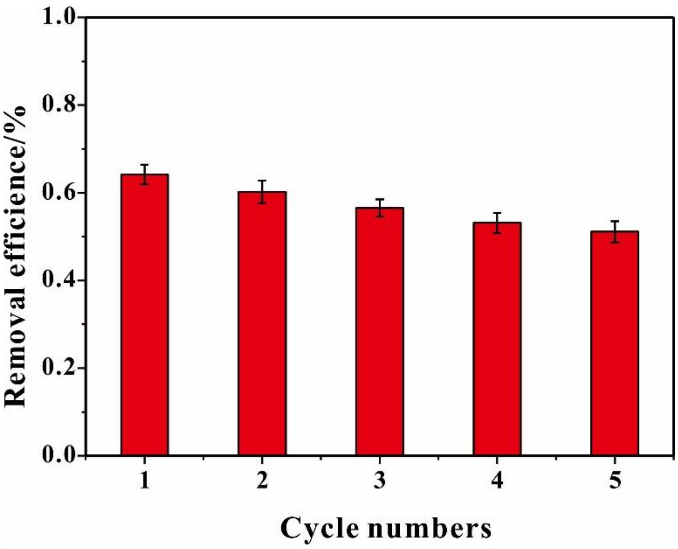

Figure S6. Removal efficiency of TOC (reaction conditions: pH 3.0; initial SMX, 20.0 mg/L; catalyst, 400 mg/L; H_2O_2, 4.0 mM).

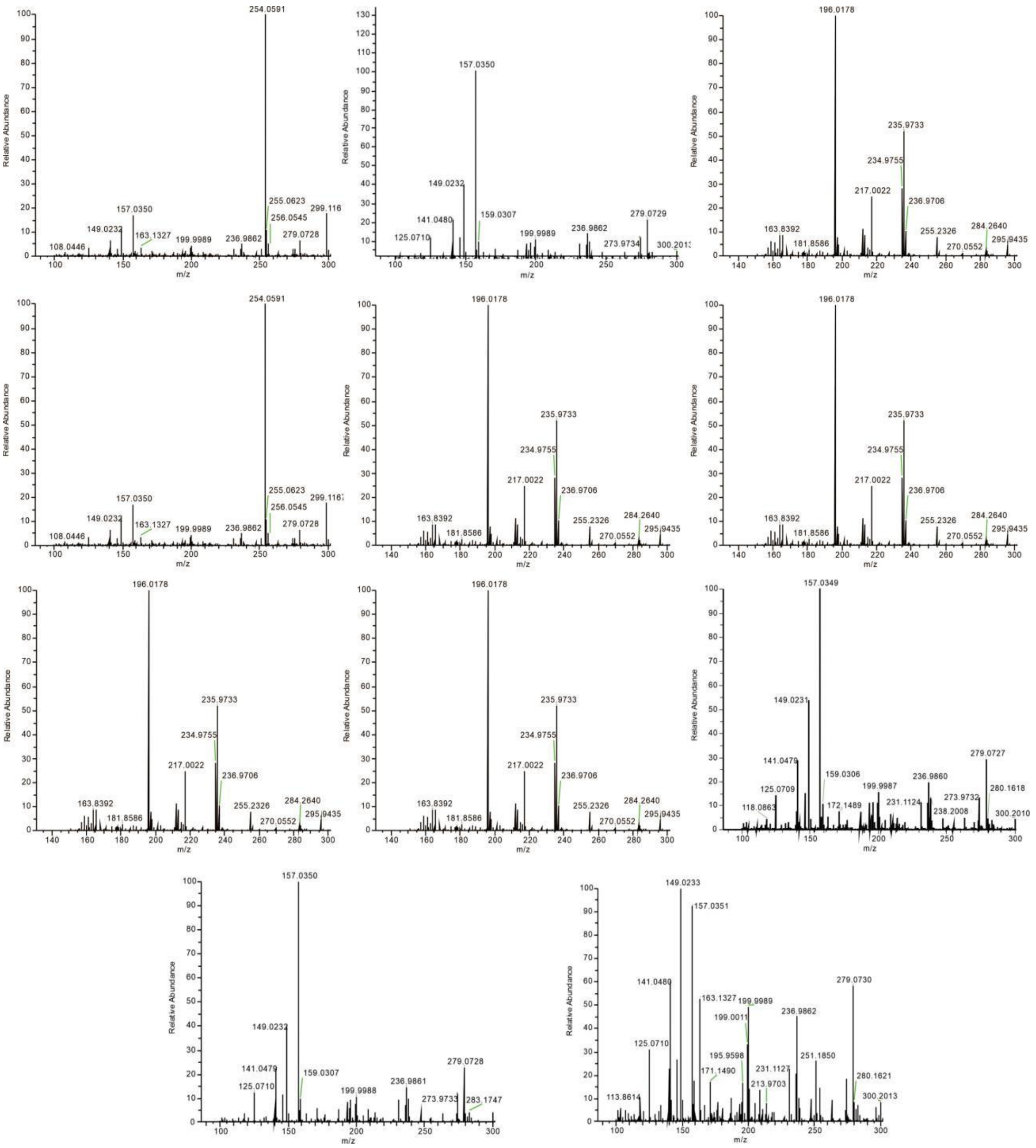

Figure S7. Liquid-mass diagram of SMX and its intermediates in photo-Fenton system.

Table S1. The chemical properties of HO-UIO-66/DE-0, HO-UIO-66/DE-300, HO-UIO-66/DE-400, and HO-UIO-66/DE-500.

**Element**	**HO-UIO-66 /DE-0**	**HO-UIO-66 /DE-300**	**HO-UIO-66 /DE-400**	**HO-UIO-66 /DE-500**
Si	39.80%	41.93%	42.08%	43.80%
O	39.11%	37.87%	37.61%	36.77%
N	14.18%	14.03%	13.85%	13.28%
C	4.36%	4.31%	4.24%	4.11%
Zr	1.45%	1.42%	1.41%	1.33%

Table S2. The structural information of the possible intermediate products.

Compounds	Formula	m/z	Proposed structure
SMX	$C_{10}H_{11}N_3O_3S$	254	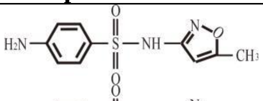
SMX 1	$C_{10}H_{13}N_3O_4S$	272	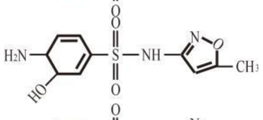
SMX 2	$C_{10}H_{11}N_3O_6S$	299	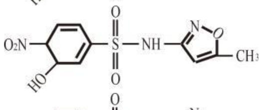
SMX 3	$C_{10}H_9N_3O_5S$	284	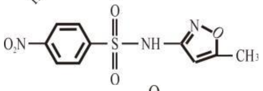
SMX 4	$C_6H_6N_2O_5S$	235	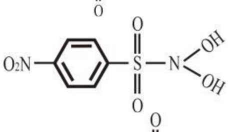
SMX 5	$C_6H_4N_2O_4S$	217	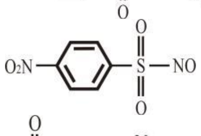
SMX 6	$C_4H_6N_2O_4S$	181	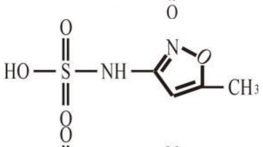
SMX 7	$C_4H_6N_2O_5S$	196	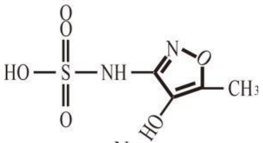
SMX 8	$C_4H_4NO_2$	113	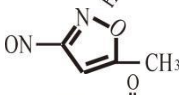
SMX 9	$C_6H_8N_2O_2S$	172	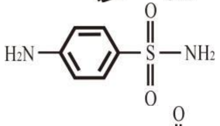
SMX 10	$C_6H_7NO_2S$	156	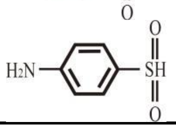
